# Isolation and Characterization of Novel Phage Displayed scFv Fragment for Human Tumor Necrosis Factor Alpha and Molecular Docking Analysis of Their Interactions

**Published:** 2018

**Authors:** Hossein Safarpour, Morteza Shahmirzaie, Elham Rezaee, Mahmood Barati, Mohammad Reza Safarnejad, Farshad H. Shirazi

**Affiliations:** a *Pharmaceutical Sciences Research Center, Shahid Beheshti University of Medical Sciences, Tehran, Iran.*; b *Department of Plant Pathology, College of Agriculture and Natural Resources, Science and Research Branch, Islamic Azad University, Tehran, Iran. *; c *Department of Pharmaceutical Chemistry, School of Pharmacy, Shahid Beheshti University of Medical Sciences, Tehran, Iran. *; d *Department of Pharmaceutical Biotechnology, School of Pharmacy, Shahid Beheshti University of Medical Sciences, Tehran, Iran. *; e *Department of Plant Viruses, Iranian Research Institute of Plant Protections, Agricultural Research, Education and Extension Organization (AREEO), Tehran, Iran.*

**Keywords:** Homology modeling, Molecular docking, Phage display, SHuffle^®^ T7 Express, Single chain variable fragment (scFv), Tumor necrosis factor alpha

## Abstract

Tumor necrosis factor alpha (TNF-α) expression amplifies to excess amounts in several disorders such as rheumatoid arthritis and psoriasis. Although, Anti-TNF biologics have revolutionized the treatment of these autoimmune diseases, formation of anti-drug antibodies (ADA) has dramatically affected their use. The next generation antibodies (*e.g.* Fab, scFv) have not only reduced resulted immunogenicity, but also proved several benefits including better tumor penetration and more rapid blood clearance. Using affinity selection procedures in this study, a scFv antibody clone was isolated from naïve Tomlinson I phage display library that specifically recognizes and binds to TNF-α. The TNF-α recombinant protein was expressed in genetically engineered *Escherichia coli* SHuffle^®^ T7 Express, for the first time, which is able to express disulfide-bonded recombinant proteins into their correctly folded states. ELISA-based affinity characterization results indicated that the isolated novel 29.2 kDa scFv binds TNF-α with suitable affinity. *In-silico *homology modeling study using ‘*ModWeb*’ as well as molecular docking study using Hex program confirmed the scFv and TNF-α interactions with a scFv-TNF- α binding energy of around -593 kj/mol which is well in agreement with our ELSIA results. The cloned scFv antibody may be potentially useful for research and therapeutic applications in the future.

## Introduction

Rheumatoid Arthritis is one of the most commonly long term inflammatory diseases. Although it generally involves the joints primarily, itis recommended as a syndrome which includes extra articular manifestations, such as rheumatoid nodules, pulmonary vasculitis or involvement, and systemic comorbidities (1). The inflammatory milieu in the synovial area is regulated by way of a complicated cytokine and chemokine network. Specialized medical interventions obviously indicate that components like TNF-α, interleukin 6, and probably granulocyte-monocyte colony stimulating factor are crucial to the procedure (2). Because inflammation is at the apex of clinical events (driving clinical symptoms, joint damage, disability, and comorbidity) (3), its reversal is a major therapeutic target; so that the physical functions of the patients improve without further squeal.

Lately, the therapeutic management of patients with RA has seen a major evolution (4). A number of biological reagents have been developed that suppress inflammation at least in a subset of patients (5, 6). Currently approved biological therapeutics for rheumatoid arthritis have four different modes of action: TNF inhibition, interleukin 6 receptor inhibition, T-cell co-stimulation blockade, and B-cell depletion. Anti-TNF therapy has resulted in major improvement not limited to patients with RA also for people that have other serious inflammatory diseases, such as Crohnʹs disease, ulcerative colitis, psoriasis, psoriatic arthritis, ankylosing spondylitis and juvenile RA. Among the TNF inhibitors, five compounds have recently been approved; one for intravenous use (infliximab) and four for subcutaneous application (adalimumab, certolizumab pegol, etanercept, and golimumab), representing close to $25 billion in sales. Etanercept is a TNF-receptor, whereas others are monoclonal antibodies or fragments of monoclonal antibodies (certolizumab) (7).

In the past decade, innovations in recombinant antibody technology have greatly facilitated the genetic manipulation of antibody fragments (8, 9). ScFv is a single chain monoclonal antibody, which has variable regions of light and heavy chains, jointed by flexible linker sequences, usually multiple glycines (10, 11). So far, about 86 scFvs are in clinical trials. 

In recent years, phage display of non-immune, human naïve scFv antibody repertoires have proven to be an important tool for the generation of highly specific antibodies (12-14). In the present paper, we describe the use of phage display technology to raise a broad panel of TNF-α specific recombinant antibodies from a human combinatorial antibody library.

## Experimental

*Large scale expression and purification of TNF-α protein*

DNA sequence corresponding to the soluble form of human TNF-α protein was codon optimized and synthesized by Bioneer Inc. (Daejeon, South Korea) for *E. coli* cell expression, and cloned into a pET28a vector (Invitrogen, Carlsbad, California, United States) containing an expression cassette with an N-terminal polyhistidine tag. Protein expression was conducted under native conditions (15) and purification was accomplished by the Ni-NTA agarose column using ion-exchange metal affinity chromatography (IMAC) method according to the manufacturer’s instructions (Qiagen, Hilden, Germany). The purity and integrity of the produced recombinant protein was analyzed by sodium dodecyl sulfate polyacrylamide gel electrophoresis (SDS-PAGE) as previously described (16).

*Panning of phage display library*

Tomlinson I naïve scFv phage library was used for the selection of specific binders against recombinant TNF-α protein. The panning procedure was carried out by immobilizing recombinant TNF-α protein (100 µg/mL) overnight onto immunotubes (Nunc, Roskilde, Denmark), which had been washed with 1X PBS buffer, blocked with 2% skim milk in 1X PBS (w/v), and incubated with phage suspension (~10^13 ^cfu) at room temperature. Phage particles with affinity for the recombinant TNF-α were eluted using 100 mM triethylamine, and amplified in exponentially growing *E. coli* TG1 cells (Source BioScience, Nottingham, United Kingdom). The panning procedure was repeated for three rounds and the total eluted phage titer was determined after each round.

*Mini-induction of scFvs*

After three rounds of panning, *E. coli* non-suppressor HB2151 cells and also TG1 cells were infected with 1 µL of eluted phage and plated on TYE agar plates containing 1% (w/v) glucose and 100 µg/mL ampicillin. In order to induce scFv production, 96 colonies of TG1 and HB2151 containing the scFv phagemids were randomly picked up and inoculated in 2xTY medium supplemented with 100 µg/mL ampicillin and 0.1% (w/v) glucose in a microtiter plate. The cultures were grown while shaking (250 rpm) at 37 °C until the OD_600_ reached about 0.9. The transcription of scFv cassette was then induced with isopropyl β-D-thiogalactopyranoside (IPTG), which was added to the culture at a final concentration of 1 mM. Shaking was continued at 200 rpm in 30 °C environment overnight. The cells were isolated by centrifugation and the supernatants were used for subsequent assays.

*Binding activity of scFv against TNF-α protein*

The binding activities of selected clones were investigated by Enzyme-Linked Immunosorbent Assay (ELISA). Briefly, About 100 µg/mL recombinant TNF-α in 1X PBS buffer was directly coated on high-binding Maxisorp microtiter plates (Nunc, Roskilde, Denmark). The wells were subsequently blocked with 2% BSA, in 1X PBS. 100 µL of scFv solutions (as described above) were then applied to the plates and incubated at 37 °C for 2 h. Bound scFvs were detected using anti c-Myc monoclonal antibody 9E10 followed by horseradish peroxidase conjugated to goat anti-mouse polyclonal antibodies. The plates were then evaluated using Biotek ELISA reader (Winooski, Vermont, United States). Positive clones were further characterized by western blotting. 

*Blotting analysis*

In Western blotting, electrophoretically separated TNF-α protein was transferred from SDS-PAGE gel to nitrocellulose membrane (0.45 µm). The membrane was blocked with PBS buffer containing 2% (w/v) skim milk. 

The scFv proteins were detected by the anti c-Myc monoclonal antibody 9E10, followed by Goat anti-mouse IgG, coupled to alkaline phosphatase (Sigma-Aldrich, St. Louis, Missouri, United States). Blots were developed using 5-bromo-4-chloro-3-indolyl phosphate (BCIP) and nitro blue tetrazolium chloride (NBT) as substrate.

*Homology modeling*

The 3D structure of the anti-TNF-α scFv was built by homology modeling based on the template crystal structure. To do so, the Anti-TNF-α scFv complex models were predicted using the structures determined by X-Ray crystallography of the different partners. The calculations were performed using the *ModWeb version r182* web server (https://modbase.compbio.ucsf.edu/modweb). Briefly, the heavy and light chain sequences were searched against antibody structure database followed by the identification of the template for framework (FRH/FRL) region.

*Molecular docking*

Hex program (version 6.0, http://hex.loria.fr/) was used to investigate the mode of interaction between the TNF-α and BSA (as negative control) with scFv. All water molecules were removed from the scFv model structure, and hydrogen atoms were added followed by minimal minimization in the presence of bound ligand using swis-pdb Viewer. The active site for docking was defined for all atoms within the 10 Å radius of the co-crystallized ligand. The correlation type was set to shape with electrostatic and subset parameters set at 0. The possible interactions were analyzed using Pymol software version 1.5.0.1 (http://pymol.findmysoft.com). The final model was selected based on the cluster size as described in the results session. 

## Results

*Protein expression and purification*

**Figure 1:**
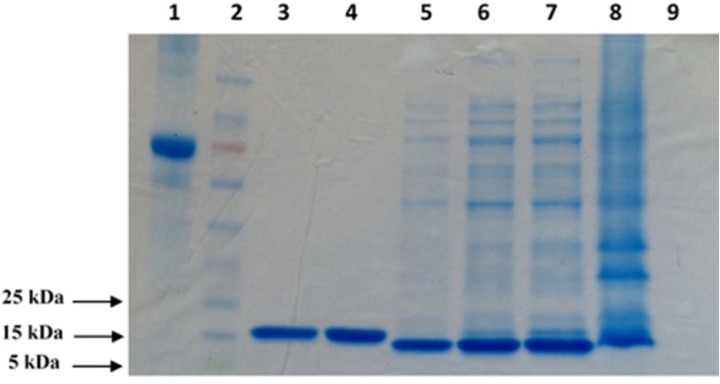
SDS–PAGE (12% Tris glycine–HCl gel) analysis of purified TNF-α recombinant protein. The position of the bands appears to indicate that the molecular weight of the expressed protein is approximately 17 kDa. 1: BSA (1 mg/mL); 2: Prestained protein ladder; 3: Elution 3; 4: Elution 4; 5: Post Wash; 6: Pre Wash; 7: Supernatant; 8: Pellet; 9: Pre Induction

To produce adequate amounts of protein for the panning procedure and further characterization of the selected scFv fragments, 6xHis-tagged TNF-α protein was expressed in SHuffle^®^ T7 Express. Induction of protein expression was performed by the addition of IPTG followed by bacterial cells disruption and the purification of recombinant protein on Ni-NTA-agarose column. Figure 1 shows the SDS-PAGE analysis results for the samples obtained at different steps of the expression and purification processes.

The result of SDS-PAGE confirmed the integrity of a well purified recombinant TNF-α protein product that migrated at approximately 17 kDa in reducing SDS-PAGE. The total yield of the purified protein in the culture medium was estimated to be about 700 µg/mL (Figure 1).

**Table 1:** Enrichment of TNF-α -specific scFv fragments through three rounds of phage display library panning.

**Round of panning**	**Input phage**	**Output phage**
1	10^13^	4.8 × 10^4^
2	10^13^	5.9 × 10^5^
3	10^13^	8.6 × 10^6^

**Figure 2:**
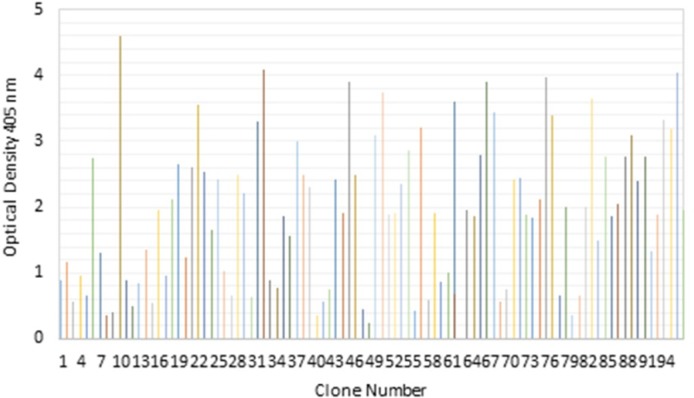
Screening of selected clones from the Tomlinson I scFv library in soluble ELISA. Binding activities of 96 randomly selected soluble scFv to TNF-α protein after the third round of panning were revealed by direct ELISA

**Figure 3:**
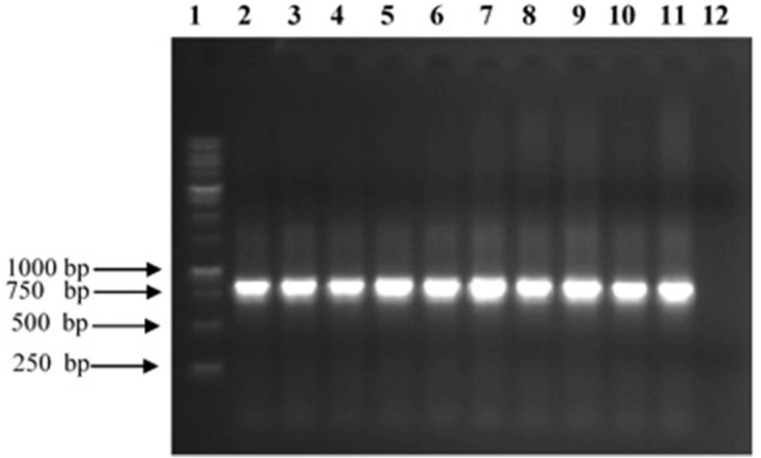
Analysis of PCR products of TNF-α binder clones from Tomlinson I phage display library. The 10 scFv genes were amplified by specific pHEN primers and analyzed on a 1% (w/v) agarose gel. 1:1 kb ladder; 2–11: selected clones reacting with TNF-α; 12: Negative control

**Figure 4.**
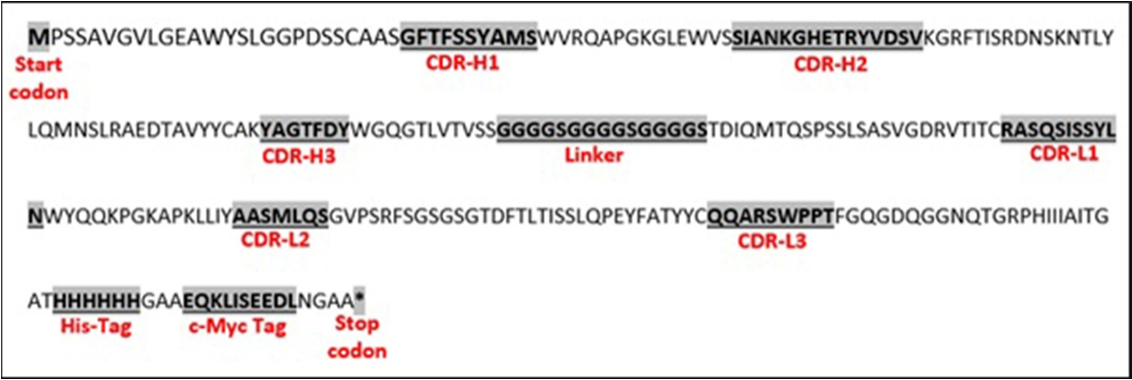
Deduced amino acid sequence (including depiction of CDRs, His-tag and c-Myc tag sections) of scFv with binding specificity to TNF-α isolated from the Tomlinson I library

**Figure 5:**
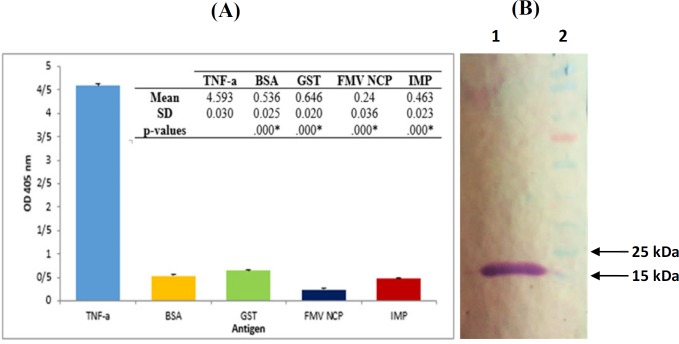
Characterization of TNF-α-scFv1. (A) Specificity of TNF-α-scFv1. ELISA plates were coated with 100 µg/mL of TNF-α, BSA, GST, IMP and FMV NCP and incubated with TNF-α-scFv1 solution. Subsequent steps were conducted as described in the text. Data are means ± SD from several independent experiments. *: *p < *0.05; significant difference from scFv1-TNF-α (one-way ANOVA, followed by Tukey’s HSD test). (B) Western blot analysis of bacterial expressed TNF-α-scFv1. Periplasmicly expressed scFv was used as the primary antibody for binding to recombinant TNF-α which was blotted onto nitrocellulose membrane. 1: 17 kDa band of TNF-α; 2: PageRuler Prestained Protein Ladder (Thermo Fisher Scientific, USA

**Figure 6.**
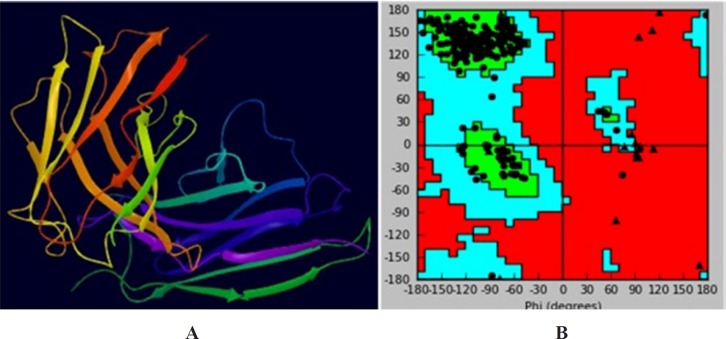
Three-dimensional homology model of scFv. (A) Three dimensional model of scFv of TNF-α. (B) Ramachandran plot of the scFv model

**Figure 7:**
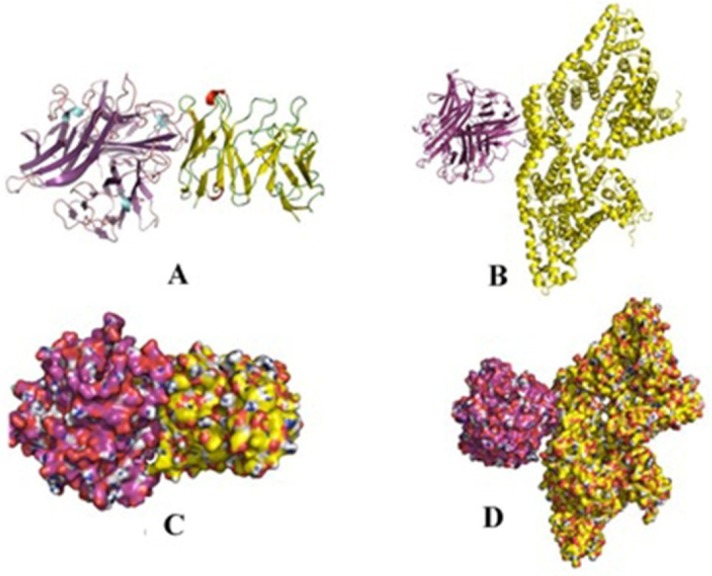
Comparison of 3D binding poses of docking complex of scFv for TNF-α and BSA. (A and C) The cartoon and surface 3D binding poses of TNF-α (purple)-scFv (yellow), respectively; (B and D) The cartoon and surface 3D model binding poses of scFv (purple)-BSA (yellow), respectively

**Figure 8:**
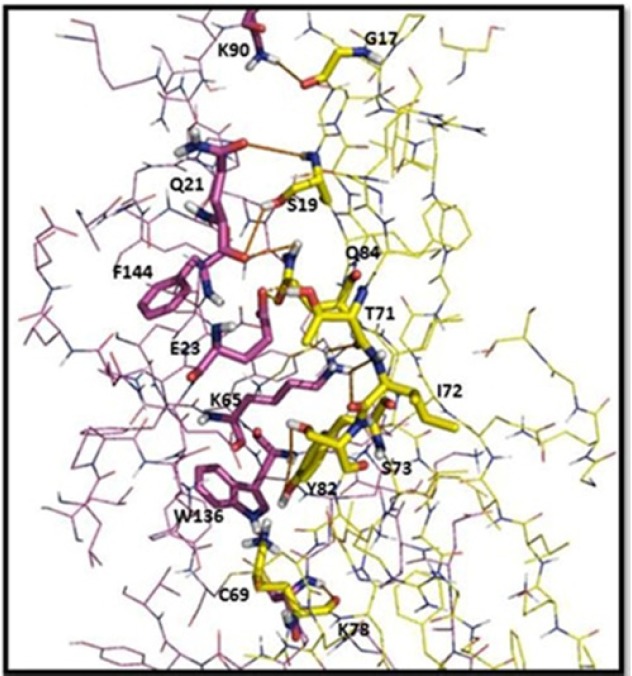
Intermolecular interaction analyses of the scFv (yellow) with TNF-α (purple). Amino acid residues involved in the interactions with the scFv are labeled, and the hydrogen bonds are shown as red lines. The distances shown are in Å

*Phage display scFv selection*

Three rounds of panning were accomplished using Tomlinson I scFv library with approximately 10^13 ^cfu of phages/round. The panning process was conducted by immobilization of the recombinant TNF-α protein onto immunotubes. The population of the eluted specific phages was evaluated following each round of panning. Table 1 represents the TNF-α -specific scFv fragments enrichment during the panning processes. A growing titer of the eluted phages in each subsequent round of panning indicated that the number and the specificity of the eluted phages had increased and that they were strongly capable of binding to the TNF-α protein.

After the third round of panning, 96 colonies were randomly selected and their binding activities against TNF-α were investigated by ELISA. Following the application of recombinant TNF-α, 10 clones with positive activity against purified recombinant TNF-α protein were selected from scFv Tomlinson I library as is shown in Figure 2. 

*Sequencing of scFv genes and blotting*

The scFv encoding regions of positive clones were PCR-amplified (834 bp) and subjected to fingerprinting analysis (Figure 3). To confirm the existence of full length V_H_ and Vκ inserts, Plasmid extraction was conducted from each colony with positive result in ELISA experiment and used for sequence analysis using specific primers for pHEN vector. By using readily available free web based tools: (http://web.expasy.org/translate/; https://blast.ncbi.nlm.nih.gov/Blast.cgi?PAGE=Proteins andwww.ebi.ac.uk/Tools/clustalw2/index.html), the translation of the DNA sequences into amino acid sequences and subsequent protein alignment were accomplished. The results confirmed that all positive clones obtained in the panning process contained the same sequence, namely TNF-α-scFv1 (predicted mass of 29.2 kDa; predicted PI of 7.1) (GenBank accession number KY072973). Alignment analysis with the available sequences in the IMGT database (http://www.imgt.org/IMGT_vquest/share/textes/) showed that the V_H_ and Vκ fragments of TNF-α-scFv1 were members of different groups as is shown in Figure 4.

*Binding characteristics of the selected scFv*

To investigate on the cross reactivity of scFv with other proteins, TNF-α-scFv1 solution (as described above) was used to detect the recombinant TNF-α protein as well as BSA, Glutathione-S-transferase (GST), Immunodominant Membrane Protein (IMP), and Fig Mosaic Virus Nucleic Capsid Protein (FMV NCP) using ELISA. According to Figure 5A, no cross reactivity of the prepared scFv with other negative control proteins was detected. Complementary Western blot analysis was performed to further confirm the specificity of the TNF-α-scFv1 against recombinant TNF-α as is shown in Figure 5B, with a distinct band around 17 kDa for the purified protein.

*Homology model building and validation*

Anti-TNF-α scFv was applied to the *ModWeb version r182* web server. The PDB file 5B3N with the lowest DOPE and the highest GA341 assessment scores was selected (Figure 6A). Quality evaluation of the model structure was performed using Ramachandran plot obtained from swis-pdb Viewer. Figure 6B indicates that about 91.1% of the amino acid residues are in the most favored regions and only 1.9% in the disallowed regions. The unfavorable residues were mainly located in the (Glycine_4_Serine_3_) linker region of the model, which had no appropriate crystal structure in the homology model template. These results showed that the scFv model was suitable for the molecular docking study.

*Docking of TNF-α and BSA with scFv*

To visually evaluate the interaction of the scFv with TNF-α, the TNF-α and BSA molecules were docked onto scFv using Hex program. The models with the lowest energy docking were selected from the resulting docking models. According to the docking results, the binding energy of scFv was estimated to be −593 kj/mol when fused to TNF-α. This is approximately 3.5 fold stronger than that of scFv-BSA (−165 kj/mol). These results are in agreement of the ELISA experiments. The final configuration of the docking complex of scFv-TNF-α is shown in Figure 7 which reveals the binding of TNF-α molecule to the gap between VL and VH domains of scFv. As shown in Figure 8, the binding domain is mainly formed by: K78, C69, Y82, S73, I72, T71, Q84, S19, and G17. Furthermore, the hydrogen bond interaction analyses revealed that the complex was stabilized by nine intermolecular hydrogen bounds.

## Discussion

The existing evidence suggests that TNF-α is involved in the pathways of inflammatory responses (17). It has been shown that targeting of TNF-α may suppress inflammation, at least in a subset of patients (5, 6). Anti TNF-α monoclonal antibodies, due to their high specificities, are generally used in clinical and diagnostic fields. So far, five anti-TNF-α therapeutic agents are approved by both the Food and Drug Administration (FDA) and European Medicines Agency (EMA) for the treatment of rheumatoid arthritis. Despite the advantages of these antibodies, some patients cannot benefit from such treatments. In some individuals, formation of anti-drug antibodies (ADA) as an immune response is triggered following the introduction of these biologic agents (18, 19). In comparison to these full size antibodies, next generation antibodies (*e.g.* Fab, scFv) not only have reduced the immunogenicity, but also have several benefits including better tumor penetration, more rapid blood clearance and lower retention times in non-target tissues (10).

As a powerful engineering tool, antibody phage display has many advantages in the selection of antibodies including library size (>10^10^), robust and well-established *E. coli* expression system, scalability, short panning cycles and cost-effectiveness (12).

In the present study, we described the use of Tomlinson I human single fold synthetic naïve phage display single chain antibody fragment library in phagemid/scFv format-fused to the pIII minor coat protein of M13 bacteriophage to identify small, fully humanized antibodies against TNF-α. For the evaluation of our results, the efficiencies of binder phages were measured as the fraction of input phages that bound to the TNF-α. The quantification of phages was performed by tittering them after each round of the bio-panning, with the input phage concentration being kept high during the rounds of selection enrichment (20, 21). The results obviously showed that the isolation and enrichment of TNF-α binding phages were accomplished. After three rounds of bio-panning, small scale production of soluble scFv antibody fragments could be conducted in the TG-1 or HB2151 *E. coli* strains according to the Tomlinson library protocol. The expressed scFvs could then be evaluated using an ELISA system. Our results showed that 2 out of 10 positive clones (Average OD~4.3) were expressed in HB2151 and the other 8 clones (Average OD~3.1) in TG-1 which is in agreement with Tomlinson library protocol. Based on this protocol, expression of soluble scFv in TG-1 strain, which is able to suppress the termination and to introduce a glutamate residue at these positions may result in more positive clones. Co-expression of the scFv-pIII fusion, as a consequence of this suppressed termination, tends to lower the overall levels of scFv expression even in clones where there are no TAG stop codons in the scFv itself. To solve this problem, non-suppressor HB2151 strain was infected with selected phages. ScFv genes that did not contain TAG stop codons yielded higher levels of soluble scFv than those in TG-1. On the other hand, those that contained TAG stop codons would not produce any soluble scFv. 

Aliazadeh *et al.* developed scFv specific antibodies against TNF-α using Tomlinson I library expressed in BL21 *E. coli* strain (22). Since a correct folding of the recombinant protein is highly important in antibody selection, here, we expressed TNF-α recombinant protein in SHuffle^®^ T7 Express which has recently been widely used for the production of recombinant proteins. SHuffle^®^ T7 Express is an *E. coli* engineered specifically for the expression of disulfide-bonded proteins. In this strain, glutaredoxin reductase and thioredoxin reductase genes are deleted (Δgor ΔtrxB). At the same time a version of the disulphide band isomerase DsbC which lacks its signal sequence is expressed in the cytoplasm. DsbC isomerizes mis-oxidized substrates into their correctly folded state and greatly enhances the fidelity of disulphide bond formation (23). We have recently Published a paper related to ATR-FTIR characterization of secondary structure of recombinant TNF-α protein which was expressed in both BL21 and SHuffle^®^ T7 Express systems. The result of our study has clearly indicated that the secondary structure of the studied TNF-α protein especially of the β-Sheets and β-Turns in SHuffle^®^ T7 Express were significantly different from those in BL21. These changes increased the structural similarity of the recombinant protein to the eukaryotic expression pattern which may consequently correct the folding (24). 

Homology modelling and molecular docking are two appropriate computational methods to study protein–ligand interactions. Here, Web based server ‘*ModWeb*’ was used forthe scFv docking studies. PDB file of 5B3N molecular model was used as input in the antigen-antibody docking algorithm. Thirty-three models were generated by the Hex program which was the highest ranked model selected for analysis. Docking results of scFv-TNF-α showed a binding energy of −593 kj/mol, which was approximately 3.5 fold stronger than that of scFv-BSA (−165 kj/mol). Furthermore, the results showed that the binding domain was mainly formed by K78, C69, Y82, S73, I72, T71, Q84, S19 and G17 amino acid residues. 

We have, therefore, successfully isolated novel scFv clone variants from a naïve or single-pot library with specific binding affinity to TNF-α. In order to achieve high yield expression (due to low level periplasmic expression of this sequence), the sequence which encodes this identified anti-TNF-α scFv antibody can be sub-cloned into an expression vector under the control of a strong T7 promoter (*e.g.* pET series) and then be transformed to the expression strains (*e.g.* SHuffle^®^ T7 Express). 
